# Reduction in residual cyantraniliprole levels in spinach after various washing and blanching methods

**DOI:** 10.3389/fnut.2022.948671

**Published:** 2022-07-28

**Authors:** Minsoo Park, Hyeonjun Kim, Myungheon Kim, Moo-hyeog Im

**Affiliations:** Department of Food Engineering, Daegu University, Gyenogsan, South Korea

**Keywords:** cyantraniliprole, washing, blanching, pesticide residue, spinach

## Abstract

Pesticides are used to protect crops from pests and diseases. However, as many pesticides are toxic to humans, it is necessary to assess methods that can remove pesticide residues from agricultural products before human consumption. Spinach is consumed immediately after a relatively simple washing and heating process in the Republic of Korea. Cyantraniliprole is used as a systemic insecticide during spinach cultivation, which means it might remain in the crop after processing. Consequently, it is important to assess whether residues can be reduced to levels that are harmless to the human body after processing. This study investigated lowering the residual cyantraniliprole levels in spinach after washing and blanching. The amount of cyantraniliprole residue in the spinach samples sprayed with cyantraniliprole during cultivation was analyzed using ultrahigh-performance liquid chromatography-tandem mass spectrometry (UHPLC–MS/MS). The time of each washing and blanching method was set at 1, 3, and 5 min. The residual levels of cyantraniliprole decreased by 15.1–54.6% and 60.1–93.5% based on the washing and blanching methods employed. The most effective washing method to lower residual cyantraniliprole levels was steeping with a neutral detergent, resulting in cyantraniliprole reduction by 42.9–54.6%. When spinach was blanched after steeping washing with a neutral detergent, the largest removal rates of 77.9 and 91.2% were observed after 1 and 3 min of blanching, respectively. Blanching for 5 min after steeping and running washing exhibited the highest reduction rate of 93.5%. Therefore, a considerable amount of cyantraniliprole residue in spinach could be removed by washing or blanching. Based on the results of this study, blanching after steeping washing can be implemented as an effective method of lowering pesticide concentrations in spinach and other crops, thereby reducing their potential toxicity to humans upon consumption.

## Introduction

Spinach is an annual crop belonging to the *Chenopodioideae* subfamily; it is an alkaline vegetable rich in various vitamins, iron, and calcium (1). In the Republic of Korea, the cultivation period of spinach is decided based on the variety; Chinese spinach is sown in autumn, and English spinach is sown in spring and summer. Hybrid spinach with the characteristics of both Chinese and English spinach is cultivated throughout the year (2). In 2019, spinach production in the Republic of Korea was 70,844 tons, with Gyeonggi-do, Gyeongsangnam-do, and Jeollanam-do producing 24,196 (~34%), 15,873 (~22%), and 8,038 (~11%) tons, respectively (3). The average daily intake of spinach in the Republic of Korea in 2018 was 5.24 g/day per person; this was the 15th highest among all vegetables consumed in the Republic of Korea (4). Pesticide usage in spinach is essential because it is susceptible to diseases, such as mosaic and fusarium wilt, and pests, such as turnip moth and *Spodoptera litura* (5). Consequently, when pesticides are not used, the productivity and quality of crops decrease (6). However, as pesticides are toxic, spray standards and pesticide residue management should be strictly implemented (7). An analysis of hazards in domestic agricultural products—conducted by the Ministry of Food and Drug Safety (MFDS), Republic of Korea—revealed that 24 pesticides exceeded the maximum residue limit (MRL) in spinach (8). The patterns of residual pesticides vary depending on their physical and chemical properties, crop types, pesticide formulations, spray method, and environmental conditions. Sufficient knowledge and understanding of pesticides are required for their use (9). Removal of pesticide residues to acceptable levels through washing and blanching technologies may be a viable approach to enhance public health by minimizing ingestion of toxic contaminants. In a consumer perception survey regarding factors that can harm agricultural safety, pesticides were voted as the highest threat (45.7%), followed by heavy metals (31.8%) and chemical fertilizers (7.3%) (10). A survey on food safety hazards reported pesticides to be one of the hazards affecting human health. These surveys indicate that consumers are concerned about pesticide use and its associated detrimental effects (11).

To provide safe agricultural products to consumers, the Korean government implements good agricultural practices (GAPs) to manage the use of pesticides that are possibly retained during the harvesting, storage, packaging, processing, and distribution of agricultural products (12). In addition, the MRL is established for each agricultural product to a level that does not affect the human body, even if pesticides retained in agricultural products are consumed every day for the rest of the person's life (13). In the case of pesticides without a set MRL, the positive list system that applies the uniform standard (0.01 mg/kg) is taken into consideration to safely manage pesticide use at the level of non-detection (14). Pesticide residues are assessed in agricultural products that are imported, distributed, and produced during their raw material stage. However, people consume agricultural products in the form of processed foods that are washed and processed (15). As the amount of most pesticide residues in agricultural products can be reduced by pyrolysis and volatilization by washing and heating during the processing stages, it is very important to determine the residual level or pesticide properties after processing (16).

Among the pesticides used in spinach, insecticides include abamectin, chlorfenapyr, tebufenozide, cyantraniliprole, and fungicides, including azoxystrobin, pyraclostrobin and cyazofamid (17). In the Republic of Korea, the MRL has been set for 124 types of pesticides in spinach, and the MRL for cyantraniliprole use in spinach is set at 3.0 mg/kg (18). Cyantraniliprole is a diamide insecticide that passes from the xylem of crops, penetrates the leaf layer and roots, and causes muscle paralysis, coma, and lethargy in pests (19, 20). Diamide pesticides are safe and selective pesticides that exhibit high insecticidal activity and low toxicity to mammals (21). Cyantraniliprole is a systemic insecticide that has medium lipophilicity (log K_ow_ of 1.94), has a low water solubility of 14.2 mg/L and is non-volatile (V_p_ of 5.13 × 10^−12^ mPa at 20°C). These properties could be major indicators to understand the reduction of residual pesticides in agricultural products (22–24).

In the Republic of Korea, various studies on cyantraniliprole and spinach have been conducted, e.g., the development of an analytical method for cyantraniliprole residues in Welsh onion (20), residual properties and risk assessment of cyantraniliprole in some minor crops (21), and evaluation of cyantraniliprole residues observed in lettuce, spinach, and radish (25). A study on spinach sprayed with chlorpyrifos revealed a decrease of 33.4% in the pesticide residue when spinach was immersed twice in tap water for 30 s and a decrease of 57.6% when spinach was blanched without washing for 1 min (26). A study was performed on the change in the residual level of triazole fungicides during the cultivation and cooking of spinach; spinach sprayed with metconazole showed a reduction in the residue by 78.4% when it was immersed in tap water for 1 min and washed (27). After washing and blanching of lettuce, azoxystrobin residual levels were reduced by 38.9–75.3% and 73.6%, respectively (28). Based on these studies, it is confirmed that washing and blanching are effective in lowering residual pesticides. To the best of our knowledge, studies assessing the reduction in cyantraniliprole residue levels in spinach after washing and blanching are non-existent in the Republic of Korea.

Unlike other agricultural products, spinach is consumed immediately after a relatively simple washing and heating process in the Republic of Korea. Moreover, cyantraniliprole is a systemic insecticide, which means it might not easily be removed even after processing. Therefore, it is important to accurately determine the reduction in levels of residual cyantraniliprole introduced by washing and heating and whether it can be removed to an extent where it is harmless to the human body. This study focused on the reduction of residual cyantraniliprole in spinach according to practical washing and blanching methods at home and identified the most effective washing and blanching method. The reduction in the residual pesticide during washing and blanching may differ depending on the amount of water, washing and blanching time, and use of detergent. Accordingly, various washing and blanching methods are needed to determine how to effectively lower residual cyantraniliprole. In this study, we aimed to investigate the reduction in cyantraniliprole residue levels in spinach after subjecting it to various washing and blanching methods.

## Materials and methods

### Materials

Cyantraniliprole (98.7%), formic acid (≥98%), sodium hydrogenate sesquihydrate (99%), sodium citrate (99%), magnesium sulfate (99.5%), and sodium chloride (99.5%) were from Sigma–Aldrich (St Louis, MO, USA), acetonitrile and methanol (both HPLC grade) were from Fisher J. T. Baker (Center Valley, PA, USA), and primary secondary amine (PSA) was purchased from Agilent Technologies (Santa Clara, CA, USA).

### Pesticide application

This experiment was performed in Chilgok, Gyeongsangbuk-do Province in the Republic of Korea. Furthermore, our protocol was designed in accordance with the GAPs of the country. Cyantraniliprole 5% DC (TORICH^®^, NongHyup Chemical, Seongnam, Republic of Korea) was diluted 1,000 times (200 L/1,000 m^2^) and sprayed twice on spinach 14 days before harvesting. The spinach samples were then collected. Samples were processed using a predetermined washing and blanching method, homogenized with a household mixer (Grinmic gold-DA10000G, DAESUNG ARTLON, Paju, Republic of Korea), and frozen at −20°C until analysis.

### Washing and blanching methods

Various washing and blanching methods were employed to assess the reduction in residual cyantraniliprole levels in spinach. With respect to the washing methods, the wash time was set at 1, 3, or 5 min referring to household wash to assess the reduction in the residual levels (29). The following washing methods were used:

1. Running washing (RW): spinach (150 g) was washed for 1, 3, or 5 min while lightly shaking with running water at a flow rate of 2 L/min. The flow rate was selected within the range that does not damage spinach leaves.
2. Steeping washing (SW): spinach (150 g) was steeped in 3 L water for 1, 3, or 5 min while stirring lightly.
3. Steeping and running washing (SRW): spinach (150 g) was steeped in 3 L water for 1, 3, or 5 min while stirring lightly and washed for 3 min while lightly shaking with running water at a flow rate of 2 L/min.
4. Running washing with neutral detergent (RWND): Three milliliters of neutral detergent (EVERMIRACLE®, EM, Jeonju, Republic of Korea) were mixed with 3 L of water (1 mL/L). Spinach (150 g) was steeped in this mixture, lightly stirred for 1, 3, or 5 min and then rinsed with running water at a flow rate of 2 L/min for 5 min to remove all neutral detergent.
5. Steeping washing with neutral detergent (SWND): Three milliliters of neutral detergent were mixed with 3 L water (1 mL/L), and spinach (150 g) was steeped in this mixture and lightly stirred for 1, 3, or 5 min and then steeped three times in the same amount of fresh water for 3 min to remove all neutral detergent.

The washed spinach was placed in a strainer and dried at room temperature for 4 h until no visible water was present on the surface of the leaves. Following this, the spinach was homogenized using a household mixer, and dry ice was added to avoid pyrolysis of the pesticide. Approximately 20 g of dry ice was placed in the mixer, and then 150 g of spinach was added. The sample was subsequently ground until it became a form of powder and then stored at −20°C.

With respect to the blanching methods, the blanching time was set at 1, 3, and 5 min according to the study of Ling (30) to assess the reduction in the residual pesticide levels. The following blanching methods were used:

1. Blanching without washing (BW): spinach (150 g) was added to 1.5 L boiling water and blanched for 1, 3, or 5 min.
2. Running washing and blanching (RB): spinach (150 g) was washed with running water at a flow rate of 2 L/min for 3 min, added to 1.5 L boiling water and blanched for 1, 3, or 5 min.
3. Steeping washing and blanching (SB): spinach (150 g) was steeped in 3 L tap water for 3 min while stirring lightly, added to 1.5 L boiling water and blanched for 1, 3, or 5 min.
4. Blanching after steeping and running washing (BSR): spinach (150 g) was steeped in 3 L tap water for 3 min while stirring lightly and washed with running water at a flow rate of 2 L/min for 3 min. Then, the spinach samples were added to 1.5 L boiling water and blanched for 1, 3, or 5 min.
5. Blanching after running washing with neutral detergent (BRND): Three milliliters of neutral detergent were mixed with 3 L tap water (1 mL/L), and spinach (150 g) was steeped in this mixture for 3 min and rinsed by lightly stirring with running water at a flow rate of 2 L/min for 5 min to remove all neutral detergent. The washed spinach was added to 1.5 L boiling water and blanched for 1, 3, or 5 min.
6. Blanching after steeping washing with neutral detergent (BSND): Three milliliters of neutral detergent were mixed with 3 L tap water (1 mL/L), and spinach (150 g) was steeped in this mixture for 3 min and steeped in the same amount of fresh water three times for 3 min to remove all neutral detergent. The washed spinach was added to 1.5 L boiling water and blanched for 1, 3, or 5 min.

The blanched spinach was placed in a strainer and dried at room temperature for 4 h until no visible water was present on the surface of the leaves. Following this, the spinach was homogenized and stored at −20°C.

### Analysis of the pesticide residue

The pesticide residues in the spinach samples were analyzed using the second protocol described in the multiresidue methods of the Korea Food Code (31). A part of the purification process described in the protocol was modified for suitability considering the pigment and impurities in spinach. Briefly, acetonitrile (10 mL) was added to the homogenized spinach sample (10 g)—placed in a 50 mL conical tube—and the mixture was shaken at 2,000 rpm for 5 min at room temperature (~20°C). Next, 4 g magnesium sulfate, 1 g sodium chloride, 1 g sodium citrate, and 0.5 g sodium hydrogen citrate sesquihydrate were added to the mixture, followed by shaking at 2,000 rpm for 5 min at ~20°C. Then, the mixture was centrifuged at 4,160 × g for 10 min at 4°C. One milliliter of supernatant was collected, and 150 mg magnesium sulfate and 50 mg PSA were added to it. The mixture was vigorously shaken for 30 s and centrifuged at 2,000 × g for 5 min at ~20°C. The supernatant was filtered using a 0.2-μm syringe filter and analyzed using UHPLC–MS/MS.

### UHPLC–MS/MS analysis

An Agilent 1290 Infinity II UHPLC coupled with an Agilent 6470 triple quadrupole mass spectrometer (Agilent Technologies, Santa Clara, CA, USA) was used for the analysis. Methanol with 0.1% formic acid and deionized water with 0.1% formic acid were used as mobile phases A and B. The mobile phase gradients (A and B) started at 80:20 for 0–1 min, ramped to 30:70 for 1–7.5 min, maintained at 30:70 for 7.5–9 min, changed to 1:99 for 9–9.1 min, held at 1:99 for 9.1–15 min, ramped to 80:20 for 15–15.10 min, and maintained at 80:20 for 15.10–20 min. An Agilent Eclipse Plus C18 (100 × 2.1 mm × 1.8 μm) was used as the analytical column. The flow rate was 0.2 mL/min, the column temperature was 40°C, and the injection volume was 5 μL. The following UHPLC–MS/MS conditions were used for analyzing cyantraniliprole: electrospray ionization and positive mode using multiple reaction monitoring (MRM). The capillary voltage was 3,500 V, and the drying gas flow and temperature were 9 L/min and 300°C, respectively. The sheath gas flow and temperature were 11 L/min and 350°C, respectively. MRM transitions were used as follows. The precursor ion was 473 m/z. Three product ions with good sensitivity were selected as qualitative and quantitative ions. Product ion 284 m/z was used for quantitative analysis, and the product ion m/z 442 and 177 m/z were used for qualitative analysis.

### Method validation for cyantraniliprole

The cyantraniliprole standard was dissolved in acetonitrile to prepare a stock solution with a concentration of 1,000 mg/L. The working solutions were prepared by diluting the stock solutions to a concentration of 100 mg/L using acetonitrile. The working solution of the cyantraniliprole standard was diluted with the spinach sample matrix to concentrations of 0.001, 0.003, 0.005, 0.010, 0.030, 0.050, and 0.100 mg/L to plot matrix-matched calibration curves. To verify the reproducibility, the cyantraniliprole standard solution with a concentration of 0.030 mg/L was continuously injected into the UHPLC–MS/MS system 10 times to assess the coefficient of variation of retention time and m/z in the chromatogram. The limit of quantification (LOQ) was calculated by considering the minimum detection mass (ng), sample weight (g), injection volume (μL), and final solution volume (mL). In addition, the recovery test was conducted with blank samples to validate the analytical method. The spinach sample was mixed with cyantraniliprole at three different concentrations, i.e., LOQ (0.003 mg/kg), 10 × LOQ (0.030 mg/kg), and 50 × LOQ (0.150 mg/kg).


$$\begin{array}{l} LOQ\;\left(\frac{mg}{kg}\right)=\\ \frac{minimum\;detection\;mass\;\left(ng\right)\times final\;solution\;volume\;\left(mL\right)}{injection\;volume\;\left(uL\right)\times sample\;weight\;\left(g\right)} \end{array}$$

### Statistical analysis

Each sample analysis was repeated three times, and the obtained data were statistically evaluated using one-way analysis of variance (ANOVA) with the Statistical Analysis System software (SAS version 9.3). Duncan's multiple range test was performed in cases of significant differences (*P* < 0.05) to confirm the differences among mean values.

## Results and discussion

### Method validation

The linearity of the matrix-matched calibration curves had an acceptable correlation coefficient *r* > 0.999. The coefficient of variation was 5.09% after 10 injections, and reproducibility was verified. The limit of detection (LOD) and LOQ used to analyze cyantraniliprole were 0.001 and 0.003 mg/kg, respectively. When the matrix samples of spinach were mixed with cyantraniliprole at concentrations of 0.003, 0.030, and 0.150 mg/kg, the recovery rate was 97.6–109%, and the coefficient of variation was 1.21–4.62% (Table 1). The analytical method was suitable for analyzing cyantraniliprole, exhibiting a recovery rate of 70–120% and a coefficient of variation within 10% of that specified in the guidelines of the Codex Alimentarius Commission (32).

**Table 1:** Recovery of cyantraniliprole in spinach.

**Compound**	**Fortification (mg/kg)**	**Recovery** ±**SD**^a^ **(%)**	**CV**^b^ **(%)**	**LOQ**^c^ **(mg/kg)**
Cyantraniliprole	0.003	97.61 ± 4.51	4.62	0.003
	0.03	96.76 ± 1.17	1.21	
	0.15	109.37 ± 1.83	1.67	

a *Standard deviation.*

b *Coefficient of variation.*

c *Limit of quantitation.*

### Lowering the residual cyantraniliprole levels in spinach after washing methods

The changes in the level of residual cyantraniliprole according to the washing time are listed in Table 2. The initial level of residual cyantraniliprole in spinach was 4.43 mg/kg. After RW, SW, SRW, RWND, and SWND for 1, 3, and 5 min, the residual cyantraniliprole levels were 3.76, 3.40, and 2.61; 3.56, 3.33, and 2.59; 3.22, 3.13, and 2.53; 2.82, 2.47, and 2.38; and 2.53, 2.10, and 2.01 mg/kg, respectively. Upon using most washing methods, the residual cyantraniliprole levels tended to decrease as the washing time increased. However, no significant differences (*P* > 0.05) were observed between 1 and 3 min SRW (residual cyantraniliprole levels 3.22 and 3.13 mg/kg; a reduction of 27.3 and 29.4%) and between 3 and 5 min RWND (2.47 and 2.38 mg/kg; a reduction of 44.2 and 46.3%).

**Table 2:** Changes in residual cyantraniliprole levels in spinach based on washing time.

**Washing time (min)**	**Raw spinach**	**Washing method**									
		**RW**		**SW**		**SRW**		**RWND**		**SWND**	
	**Residue amount (mg/kg) Mean ±SD**	**Residue amount (mg/kg)** **Mean ±SD**	**% Removed** ^1^	**Residue amount (mg/kg)** **Mean ±SD**	**% Removed**	**Residue amount (mg/kg)** **Mean ±SD**	**% Removed**	**Residue amount (mg/kg)** **Mean ±SD**	**% Removed**	**Residue amount (mg/kg)** **Mean ±SD**	**% Removed**
1	4.43 ± 0.140	3.76 ± 0.070^a^	15.1	3.56 ± 0.010^a^	19.6	3.22 ± 0.090^a^	27.3	2.82 ± 0.040^a^	36.3	2.53 ± 0.060^a^	42.9
3		3.40 ± 0.100^b^	23.3	3.33 ± 0.110^b^	24.8	3.13 ± 0.030^a^	29.4	2.47 ± 0.120^b^	44.2	2.10 ± 0.040^b^	52.6
5		2.61 ± 0.160^c^	41.1	2.59 ± 0.140^c^	41.5	2.53 ± 0.130^b^	42.9	2.38 ± 0.170^b^	46.3	2.01 ± 0.020^c^	54.6

1 *% Removed: 100 – (processing residue/raw product residue) × 100*.

*RW, running washing; SW, steeping washing; SRW, steeping and running washing; RWND, running washing with neutral detergent; SWND, steeping washing with neutral detergent*.

*Values followed by the same letter in the same column are not significantly different (P < 0.05)*.

A comparison of the residual cyantraniliprole levels in spinach according to each washing method is provided in Table 3. After 1 min of washing, the residual cyantraniliprole levels after SWND, RWND, SRW, SW, and RW were 2.53, 2.82, 3.22, 3.56, and 3.76 mg/kg, with reductions of 42.9, 36.3, 27.3, 19.6, and 15.1%, respectively. Among all washing methods, SWND was the most effective at reducing the residual cyantraniliprole levels (reduction rate, 42.9%). SW resulted in a more effective reduction in the residual cyantraniliprole levels than RW (19.6 vs. 15.1%). Ko et al. (33) showed that the diazinon reduction rate in lettuce was 33.7% after SW and 24.7% after RW; therefore, SW was more effective than RW in reducing the levels of residual diazinon. SRW (27.3%) was more effective than SW and RW (19.6 and 15.1%, respectively) at reducing the levels of residual cyantraniliprole. The reduction rates of residual cyantraniliprole levels after RWND (36.3%) and SWND (42.9%) were higher than those after RW (15.1%) and SW (19.6%). Consequently, cyantraniliprole levels tended to decrease more effectively when washed with a neutral detergent. Kwon et al. (34) reported a decrease of 3–4% in chlorpyrifos levels when lettuce was washed with tap water and a decrease of 12–31% when it was washed with a neutral detergent. Lee et al. (35) reported that the levels of chlorpyrifos-methyl sprayed on perilla leaves decreased by 44.3 and 81.5% after washing with tap water and neutral detergent, respectively. Our results are consistent with those of these studies because chlorpyrifos and cyantraniliprole are fat-soluble, and their levels were considerably reduced when a neutral detergent was used.

**Table 3:** Comparison of the residual cyantraniliprole levels in spinach based on the washing method.

**Washing method**	**Washing time (min)**					
	**1**		**3**		**5**	
	**Residue amount (mg/kg)** **Mean ±SD**	**% Removed**	**Residue amount (mg/kg)** **Mean ±SD**	**% Removed**	**Residue amount (mg/kg)** **Mean ±SD**	**% Removed**
Raw spinach	4.43 ± 0.140					
RW	3.76 ± 0.070^a^	15.1	3.40 ± 0.100^a^	23.3	2.61 ± 0.160^a^	41.1
SW	3.56 ± 0.010^b^	19.6	3.33 ± 0.110^a^	24.8	2.59 ± 0.140^a^	41.5
SRW	3.22 ± 0.090^c^	27.3	3.13 ± 0.030^b^	29.4	2.53 ± 0.130^a^	42.9
RWND	2.82 ± 0.040^d^	36.3	2.47 ± 0.120^c^	44.2	2.38 ± 0.170^a^	46.3
SWND	2.53 ± 0.060^e^	42.9	2.10 ± 0.040^d^	52.6	2.01 ± 0.020^b^	54.6

*Values followed by the same letter in the same column are not significantly different (P < 0.05)*.

After 3 min of washing, the residual cyantraniliprole levels after SWND, RWND, SRW, SW, and RW were 2.10, 2.47, 3.13, 3.33, and 3.40 mg/kg, with reductions of 52.6, 44.2, 29.4, 24.8, and 23.3%, respectively. Among all washing methods, SWND exhibited the highest reduction rate in residual cyantraniliprole levels (52.6%). The reduction rate in cyantraniliprole levels after RW (24.8%) was slightly higher than that after SW (23.3%); however, the difference was not significant. The levels of residual cyantraniliprole decreased to a greater extent after SRW (29.4%) than after RW (24.8%) and SW (23.3%). The rates of reduction in residual cyantraniliprole levels after RWND (44.2%) and SWND (52.6%) were significantly higher than those after RW (23.3%) and SW (24.8%). Therefore, cyantraniliprole levels tended to decrease more effectively when the spinach was washed with a neutral detergent (compared to when it was washed with water).

After 5 min of washing, the residual cyantraniliprole levels after SWND, RWND, SRW, SW, and RW were 2.01, 2.38, 2.53, 2.59, and 2.61 mg/kg, with reductions of 54.6, 46.3, 42.9, 41.5, and 41.1%, respectively. Among all washing methods, SWND was the most effective at reducing cyantraniliprole levels (rate, 54.6%). No significant difference was observed in the levels of residual cyantraniliprole among RW, SW, SRW, and RWND.

Based on the overall results, it can be observed that residual cyantraniliprole can be lowered considerably by washing even though its water solubility is low (14.2 mg/L). The reason is assumed to be that residual cyantraniliprole remains more abundant on the surface of spinach than in its inner tissues because cyatraniliprole is relatively non-polar (log K_ow_ of 1.94) and interacts with plant epicuticular wax on the surface (36). Tahir et al. (37) reported that although cypermethrin has low solubility in water (0.01 mg/L), the reduction rate in washed vegetables was 6–100%. Klinhom et al. (38) showed that methomyl, which is a systemic pesticide, decreased by 37.90% in leafy Chinese-Kale after washing. These studies show that pesticides that have a low solubility in water or are systemic pesticides can be lowered by washing as a result of the study. The use of detergent solution is more effective in lowering residual cyantraniliprole than tap water. Due to the surfactant component of the neutral detergent, it seems that detergent solution has more influence than tap water on the reduction of cyantraniliprole. With respect to SW and RW, SW showed a higher reduction rate than RW. The reason is presumed to be that the amount of water that touches the surface of spinach during SW is higher than that of RW.

### Lowering the residual cyantraniliprole levels in spinach after blanching methods

The changes in the level of residual cyantraniliprole according to the blanching time are shown in Table 4. After BW for 1, 3, and 5 min, the residual cyantraniliprole levels were 1.77, 1.02, and 0.800 mg/kg, with reduction rates of 60.1, 77.0, and 81.9%, respectively. This confirmed that a considerable amount of cyantraniliprole was removed just by blanching. Ryu (39) showed that when Korean cabbage sprayed with hexaconazole was blanched for 30 s without washing, the rate of hexaconazole reduction was 80.63%, i.e., more than 50% of the pesticide residue was removed only by blanching. After RB, SB, BSR, BRND, and BSND for 1, 3, and 5 min, the residual cyantraniliprole levels were 1.28, 0.610, and 0.350; 1.00, 0.630, and 0.300; 1.05, 0.400, and 0.290; 1.01, 0.490, and 0.370; and 0.980, 0.390, and 0.330 mg/kg, respectively. In most blanching methods, the residual cyantraniliprole levels tended to decrease as the blanching time increased. This is consistent with the results of Kim (40), who showed that the levels of procymidone residues in spinach decreased by 68.95–85.46% as the blanching time increased from 15, 30 s, and 1–3 min. However, no significant difference was observed in the residual cyantraniliprole levels after BSND for 3 and 5 min (0.390 and 0.330 mg/kg with a reduction of 91.2 and 92.6%, respectively).

**Table 4:** Changes in the residual cyantraniliprole levels in spinach based on blanching time.

**Blanching time**	**Raw spinach**	**Blanching time**											
		**BW**		**RB**		**SB**		**BSR**		**BRND**		**BSND**	
	**Residue amount** **(mg/kg) Mean ±SD**	**Residue amount** **(mg/kg)** **Mean ±SD**	**% Removed**	**Residue amount** **(mg/kg)** **Mean ±SD**	**% Removed**	**Residue amount** **(mg/kg)** **Mean ±SD**	**% Removed**	**Residue amount** **(mg/kg)** **Mean ±SD**	**% Removed**	**Residue amount** **(mg/kg)** **Mean ±SD**	**% Removed**	**Residue amount** **(mg/kg)** **Mean ±SD**	**% Removed**
1	4.43 ± 0.140	1.77 ± 0.080^a^	60.1	1.28 ± 0.070^a^	71.1	1.00 ± 0.080^a^	77.4	1.05 ± 0.060^a^	76.3	1.01 ± 0.010^a^	77.2	0.980 ± 0.060^a^	77.9
3		1.02 ± 0.020^b^	77.0	0.610 ± 0.030^b^	86.2	0.630 ± 0.050^b^	85.8	0.400 ± 0.030^b^	91.0	0.490 ± 0.040^b^	88.9	0.390 ± 0.050^b^	91.2
5		0.800 ± 0.080^c^	81.9	0.350 ± 0.050^c^	92.1	0.300 ± 0.020^c^	93.2	0.290 ± 0.010^c^	93.5	0.370 ± 0.030^c^	91.7	0.330 ± 0.020^b^	92.6

*BW, blanching without washing; RB, running washing and blanching; SB, steeping washing and blanching; BSR, blanching after steeping and running washing; BRND, blanching after running washing with neutral detergent; BSND, blanching after steeping washing with neutral detergent*.

*Values followed by the same letter in the same column are not significantly different (P < 0.05)*.

A comparison of the levels of residual cyantraniliprole in spinach according to each blanching method is provided in Table 5. After 1 min of blanching, the residual cyantraniliprole levels after BSND, BRND, SB, BSR, RB, and BW were 0.980, 1.01, 1.00, 1.05, 1.28, and 1.77 mg/kg, with reduction rates of 77.9, 77.2, 77.4, 76.3, 71.1, and 60.1%, respectively. Among all blanching methods, BSND was the most efficient at reducing cyantraniliprole levels (77.9%); however, no significant difference was observed among the reduction rates obtained using BSND, BRND, BSR, and SB after 1 min of blanching. Consequently, the reduction in the levels of cyantraniliprole residues was similar after 1 min of blanching after BSND, BRND, BSR, and SB. Comparing the blanching methods after washing (BSND, BRND, SB, BSR, and RB), we found that the reduction in residual cyantraniliprole levels was the lowest after RB (71.1%). This was similar to the result obtained for the washing methods where RW resulted in the lowest reduction in residual cyantraniliprole levels among all washing methods after 1 min of washing. BW resulted in a reduction rate of 60.1%, which was considerably lower than those obtained with all blanching methods. Consequently, washing before blanching was more effective than BW with respect to reducing cyantraniliprole levels.

**Table 5:** Changes in the residual cyantraniliprole levels in spinach based on the blanching method.

**Blanching method**	**Blanching time**					
	**1**		**3**		**5**	
	**Residue amount (mg/kg) Mean ±SD**	**% Removed**	**Residue amount (mg/kg) Mean ±SD**	**% Removed**	**Residue amount (mg/kg) Mean ±SD**	**% Removed**
Raw spinach	4.43 ± 0.140					
BW	1.77 ± 0.080^a^	60.1	1.02 ± 0.020^a^	77.0	0.800 ± 0.080^a^	81.9
RB	1.28 ± 0.070^b^	71.1	0.610 ± 0.030^b^	86.2	0.350 ± 0.050^bc^	92.1
SB	1.00 ± 0.080^c^	77.4	0.630 ± 0.050^b^	85.8	0.300 ± 0.020^bc^	93.2
BSR	1.05 ± 0.060^c^	76.3	0.400 ± 0.030^d^	91.0	0.290 ± 0.010^c^	93.5
BRND	1.01 ± 0.010^c^	77.2	0.490 ± 0.040^c^	88.9	0.370 ± 0.030^b^	91.7
BSND	0.980 ± 0.060^c^	77.9	0.390 ± 0.050^d^	91.2	0.330 ± 0.020^bc^	92.6

*Values followed by the same letter in the same column are not significantly different (P < 0.05)*.

After 3 min of blanching, the residual cyantraniliprole levels after BSND, BSR, BRND, RB, SB, and BW were 0.390, 0.400, 0.490, 0.610, 0.630, and 1.02 mg/kg, with reductions of 91.2, 91.0, 88.9, 86.2, 85.8, and 77.0%, respectively. Among all blanching methods, BSND was the most effective at reducing the residual cyantraniliprole levels (91.2%); however, this was not significant compared with the reduction rate obtained with BSR (91.0%). BW resulted in the lowest reduction in residual cyantraniliprole levels (77.0%) among all blanching methods. Therefore, a considerable difference was observed in the residual cyantraniliprole levels with and without washing before blanching.

After 5 min of blanching, the residual cyantraniliprole levels after BSR, SB, BSND, RB, BRND, and BW were 0.290, 0.300, 0.330, 0.350, 0.370, and 0.800 mg/kg, with reductions of 93.5, 93.2, 92.6, 92.1, 91.7, and 81.9%, respectively. It was confirmed that washing before blanching was the most effective at removing most of the cyantraniliprole residue. This was consistent with the results of a previous study (41), where the reduction rates of pymetrozine and difenoconazole were 99.5 and 100%, respectively, when water celery was washed for 2 min and blanched for 1 min. The rate of reducing cyantraniliprole levels was the highest after BSR (93.5%) among all blanching methods; however, the difference was not significant among the rates after BSR, SB, BSND, and RB. Therefore, these methods were similarly effective at removing cyantraniliprole after 5 min of blanching. BW resulted in the lowest reduction rate (81.9%) among all blanching methods.

Based on the overall results, residual cyantraniliprole appeared to be considerably lowered by blanching; nevertheless, it is non-volatile (V_p_ of 5.13 × 10^−12^ mPa at 20°C). The reason is supposed to be that residual cyantraniliprole is lowered by not only volatilization but also hydrolysis due to leaching during blanching (42). Blanching is effective in lowering residual cyantraniliprole even without washing, and it is more effective with washing. In this context, Kim et al. (43) reported that pyridaben, which has low volatility (V_p_ of 1.0 × 10^−2^ at 20°C), decreased by 85.1–90.5 in pepper leaves following washing and blanching. Consequently, to lower residual cyantraniliprole as much as possible, washing should be employed before blanching.

### Limitations of the study

This study was conducted under laboratory conditions. Consequently, the results do not represent all cases of washing and blanching spinach. Reduction of cyantraniliprole residue in spinach would vary depending on washing and blanching conditions such as the amount of water, flow rate of running water, number of washes, blanching times, type of detergent, and water temperature. Future studies should include these variables in the methodology to identify more effective measures for reducing pesticides in human foods. Furthermore, while washing with tap water is effective for polar pesticides, neutral detergents are more effective than tap water for non-polar chemicals, and the detergent solution may remain on the surface of the spinach, introducing another potential toxin to the human body. Therefore, ensuring that all detergent solution is removed after washing is important. Ascertaining the amounts of detergent that remains after washing with this solution and how much this decreases after washing may be part of a future study.

## Conclusions

The rate of reduction in cyantraniliprole levels was 42.9–54.6% after SWND, which was the most effective at reducing the levels of the cyantraniliprole residue among all the washing methods. Among the blanching methods, the highest efficiency of removing cyantraniliprole residue was observed for BSND (reduction rate of 77.9%), SB (77.4%), BSR (76.3%), and BRND (77.2%) after 1 min of blanching (though these were similar); for BSND (91.2%) and BSR (91.0%) after 3 min of blanching; and for BSR (93.5%), SB (93.2%), RB (92.1%), and BSND (92.6%) after 5 min of blanching. Since all amounts of residual cyantraniliprole after washing and blanching methods were below the MRL, residual cyantraniliprole in spinach can be removed to an extent that is harmless to the human body after washing and blanching. In summary, it was confirmed that a considerable amount of cyantraniliprole residue in spinach can be removed by washing or blanching. Blanching after steep washing was the most efficient method that removed up to 93.5% of the cyantraniliprole residue from spinach. These results can be considered a viable approach to enhance public health by minimizing the ingestion of residual cyantraniliprole. Furthermore, it can be utilized to predict the reduction tendency of residual cyantraniliprole in the food industry.

## Data availability statement

The raw data supporting the conclusions of this article will be made available by the authors, without undue reservation.

## Author contributions

MP, HK, MK, and M-hI conceptualized this study. MP, HK, and MK investigated all information needed for this study, were responsible for washing, blanching the spinach, and analyzing the cyantraniliprole residue present in the spinach samples obtained. M-hI supervised this study and reviewed and edited it. MP wrote the original draft. All authors contributed to the article and approved the submitted version.

## Funding

This research was supported by Daegu University Research Grant, 2020

## Conflict of interest

The authors declare that the research was conducted in the absence of any commercial or financial relationships that could be construed as a potential conflict of interest.

## Publisher's note

All claims expressed in this article are solely those of the authors and do not necessarily represent those of their affiliated organizations, or those of the publisher, the editors and the reviewers. Any product that may be evaluated in this article, or claim that may be made by its manufacturer, is not guaranteed or endorsed by the publisher.
