# Corrigendum: Reduction in residual cyantraniliprole levels in spinach after various washing and blanching methods

**DOI:** 10.3389/fnut.2024.1469028

**Published:** 2024-08-23

**Authors:** Minsoo Park, Hyeonjun Kim, Myungheon Kim, Moo-hyeog Im

**Affiliations:** Department of Food Engineering, Daegu University, Gyenogsan, South Korea

**Keywords:** cyantraniliprole, washing, blanching, pesticide residue, spinach

In the published article, there was an error in “Table 1. Recovery of cyantraniliprole in spinach” as published. The figure listed in the column LOQ read as 0.030. The corrected figure should read as 0.003. The corrected Table and its caption appear below.

**Table 1:** Recovery of cyantraniliprole in spinach.

**Compound**	**Fortification**	**Recovery**	**CV^b^**	**LOQ^c^**
	**(mg/kg)**	±**SD**^a^ **(%)**	**(%)**	**(mg/kg)**
Cyantraniliprole	0.003	97.61 ± 4.51	4.62	0.003
	0.03	96.76 ± 1.17	1.21	
	0.15	109.37 ± 1.83	1.67	

^a^Standard deviation.

^b^Coefficient of variation.

^c^Limit of quantitation.

In the published article, there was an error in **Materials and methods**, “*UHPLC-MS/MS analysis*.” The incorrect text reads as “MRM transitions were used as follows. The precursor ion m/z 473 and product ion m/z 284 were used for quantitative analysis, and the precursor ion m/z 442 and product ion m/z 177 m/z were used for qualitative analysis.” This should be written as “MRM transitions were used as follows. The precursor ion was 473 m/z. Three product ions with good sensitivity were selected as qualitative and quantitative ions. Product ion 284 m/z was used for quantitative analysis, and the product ion m/z 442 and 177 m/z were used for qualitative analysis.”

The authors apologize for this error and state that this does not change the scientific conclusions of the article in any way. The original article has been updated.

